# Behavioural Development of Three Former Pet Chimpanzees a Decade after Arrival at the MONA Sanctuary

**DOI:** 10.3390/ani12020138

**Published:** 2022-01-07

**Authors:** Olga Feliu, Marti Masip, Carmen Maté, Sònia Sánchez-López, Dietmar Crailsheim, Elfriede Kalcher-Sommersguter

**Affiliations:** 1 Department of Clinical Psychology and Psychobiology, Faculty of Psychology, University of Barcelona, 08035 Barcelona, Spain; 2 Research Department, Fundació Mona, 17457 Girona, Spain; martimasipgimeno@gmail.com (M.M.); recerca@fundacionmona.org (D.C.); 3 Department of Animal Rights, Barcelona City Council, C/Perez Galdós 24-26, 08012 Barcelona, Spain; carmenmategarcia@gmail.com; 4 Area of Psychobiology, Faculty of Psychology and Educational Sciences, Universitat Oberta de Catalunya, 08018 Barcelona, Spain; soniasl@hotmail.com; 5 Institute of Biology, University of Graz, Universitätsplatz 2, 8010 Graz, Austria

**Keywords:** chimpanzee, *Pan troglodytes*, activity budget, sanctuary, re-socialization, well-being, early life experience, pet and entertainment

## Abstract

**Simple Summary:**

Experiences during infancy and as a juvenile are very influential on the lives of primates into adulthood. In this sense, the living conditions of chimpanzees kept as pets or performing in circuses cannot fulfil the three basic requirements needed for these animals to develop properly: adequate functioning of the organism (physical well-being); an optimal emotional state with the absence of sensations such as fear, pain, grief or apathy (mental well-being); and the ability to express species-specific behaviours (social well-being). In this study, we compare the activity budgets of three chimpanzees approximately one-decade post-rescue, to historical activity data before their rehabilitation. We found changes in behavior patterns in accordance with the sanctuary rehabilitation objectives. All chimpanzees improved their social competence by adding new members to their social network. Vigilance behavior also declined, and time spent resting increased when living at the sanctuary. Our results support previous studies conducted with rehabilitated chimpanzees in sanctuaries and highlight the important work of dedicated professionals during the rehabilitation process for these chimpanzees housed in captivity for the rest of their life.

**Abstract:**

Chimpanzees used as pets and in the entertainment industry endure detrimental living conditions from early infancy onwards. The preferred option for ending their existence as pet or circus chimpanzees is their rescue and transfer to a primate sanctuary that will provide them with optimal living and social conditions, so that they can thrive. In this case study, we had the rare opportunity to compare the activity budgets of three chimpanzees from their time as pets in 2004 to their time living at the MONA sanctuary in 2020, after almost a decade in the centre. We found their behaviour patterns changed in accordance with the sanctuaries’ rehabilitation objectives. Resting periods increased considerably while vigilance simultaneously declined sharply. Moreover, the chimpanzees’ social competence increased as allogrooming became the predominant social behaviour, and agonistic interactions diminished even though they were living within a larger social group at the sanctuary. All three chimpanzees expanded their allogrooming and proximity networks at the sanctuary, which included new group members, but they maintained the closest relationships to those conspecifics who they were rescued with. In conclusion, these findings suggest that the sanctuary environment and social group setting made it possible for these three chimpanzees to improve their social competence and increase their well-being over time.

## 1. Introduction

According to the European Studbook for the Chimpanzee, by the end of 2014 over 1059 chimpanzees were living in captivity in Europe [1]. These chimpanzees were housed in institutions such as zoos, animal parks and sanctuaries. However, in addition to these registered individuals, there are privately owned chimpanzees, which are not registered by the EEP (European Endangered Species Programme), nor listed in any other official database. Despite bans on the import of exotic animals, and wildlife trafficking becoming illegal, international trading still exists [2], including the possession of exotic pets by private owners in several European countries [3]. Illegal wildlife trading remains one of the most profitable criminal business activities to date, with people paying enormous sums of money for infant chimpanzees [4]. Furthermore, one can still find some circuses using chimpanzees to entertain their audience across Europe. Although European circuses should comply with the Council Regulation (EC) 338/97 of 9 December 1996 on the protection of endangered species of wild fauna and flora, there is no specific legislation dealing with circuses across Europe [5]. Nineteen EU Member States have total bans on the use of wild animals in circuses. This is not the case for France, Germany, Italy and Spain, where only regional restrictions have been adopted in some but not all municipalities [6].

The vast majority of chimpanzees used in circuses, commercials and/or as pets were taken from the wild as infants where they typically experienced the killing of group members, including their mothers [2,7]. Chimpanzees kept as pets often end up in small cages when they have matured and can no longer be safely managed and controlled by their owners [8]. Traumatic early life experiences such as maternal deprivation and adverse rearing conditions affect the socio-emotional development of these orphans, producing a long-term impact which is reflected not only in their behaviour [9,10,11] but also in their personality [12,13,14]. This is not surprising, considering the similarities in the developmental trajectories of chimpanzees and humans, including the long period of dependency on their mothers. Chimpanzees are highly social beings living in complex fission-fusion societies. The infants depend strongly on their mothers in order to learn the necessary set of skills to establish their position, as well as to maintain social relationships and to navigate through complex social lives [15,16,17,18,19,20]. As such, the mothers’ presence is not only crucial to the survival of the offspring, i.e., by providing safety and food, but the mother also functions as the most important social learning partner for infants in order to develop all necessary social, emotional and cognitive skills [15,21,22]. The infants’ developmental milestone emergence of gross motor traits followed by communication, social interaction traits and fine motor traits thereafter is more or less comparable to human infants [23]. Social play with the mother and other group members is key to social development and occurs already within the first months of an infant’s life, peaking at the age of two years in males and three years in females [24], and declines sharply at adolescence [25]. Meanwhile, social grooming is the most important social tool used to establish and maintain social relationships in chimpanzees [26]. This is exhibited only rarely in infants up to their second year of life, with measurable amounts occurring after their fourth year of life [27], and mutual grooming emerging at about 38 months of age [23]. This shows that the rearing conditions during the first years of an infant’s life are of paramount importance for socio-emotional development, and adverse experiences during this period might have lifelong consequences, manifested even in socially reared zoo chimpanzees who were caught from the wild [28]. The first years of an infant’s life are important for the acquisition of ecological skills as well. Object manipulation in the form of play and exploratory behaviour, for instance, was found to occur frequently at early ages in wild-living chimpanzees but declines gradually with age, and stops in older individuals once they become habitual tool users [29].

Thus, the behavioural development of former pet and entertainment chimpanzees are likely to be impaired by the traumatic events and adverse living conditions suffered during their former lives. Although the average life span is usually shorter, chimpanzees may live up to over 60 years in captivity [15]. In addition to efforts towards improving legislations and strengthening the law enforcement efforts regarding poaching and wildlife trafficking, the detection, confiscation and rescue of privately owned chimpanzees is essential to end the existence of chimpanzees being held as pets, and is one of the primary objectives of primate sanctuaries [30,31,32]. In these sanctuaries, which by definition provide long-term or lifetime care [33], professionals work on the rehabilitation and re-socialization of chimpanzees by offering them a safe haven where they spend the rest of their lives with conspecifics in social groups. Sanctuaries strive to improve the individual’s well-being by providing an environment and conditions for these animals to heal and rehabilitate in species-adequate habitats, which promote species-specific behaviours [34].

Once a pet or entertainment chimpanzee has been located, it often still takes years to transfer this individual to a sanctuary due to the lack of clear/specific laws, the indifference/neglect by administrative authorities, and the efforts made by the owners to stall or prevent the rescue activities. Once the administrative hurdles have been solved and the chimpanzee can be safely transported to its new home at the sanctuary, he will probably experience an at least mildly stressful habituation phase, but will become gradually familiar with the new environment and living conditions.

The rehabilitation of former pet and entertainment chimpanzees is a long-lasting and complex process [35]. The well-being of incoming chimpanzees is expected to improve after a relatively short amount of time by providing an adequate physical and social environment [36,37], and a high level of attention by the professional care staff [38,39]. However, their physical and mental health most likely requires special attention for the rest of their lives [31,40]. The re-socialisation process, consisting of the introduction of and functioning within a social group of conspecifics, is one of the riskiest parts of rehabilitation. Caregivers have reported an increased likelihood of wounding incidents, especially during the very early stages of the process. However, it is also one of the most crucial steps to increase the well-being of these animals. The presence of and access of these animals to social conspecifics provides additional stimulation, and potentially socially challenging events. Most importantly, it allows them to display the behavioural repertoire of a chimpanzee. Some former pet and entertainment chimpanzees may have had periods in their life where they were housed with other conspecifics and therefore acquired some basic social skills. Many others arrive at sanctuaries without the necessary set of skills or experience to interact with other chimpanzees. Learning to interact and live within a social group is a key element to successful rehabilitation. Professional caregivers at sanctuaries have the knowledge and resources to support traumatized chimpanzees in helping them to overcome their individual impairments and slowly guiding them to a life within a social group.

The monitoring of rescued chimpanzees and the evaluation of their rehabilitation progress start upon arrival at the sanctuary, i.e., post-rescue. In this case, however, we had the rare opportunity to obtain behavioural data pre-rescue on the activity and association of three privately owned pet chimpanzees. These three orphans were imported from Africa to Spain after being caught in the wild in the 1980s and 1990s. All three individuals were exposed to close human contact, were held predominately in species-inappropriate conditions, and were housed in facilities which limited their ability to exhibit the full range of species-specific behaviours.

The main objective of this case study was to evaluate changes in the behaviours of these three chimpanzees by comparing their activity budgets and associations pre-rescue to those after living at the sanctuary for 10 years. Rather than only reviewing their general activity patterns, we also focused on their social interaction patterns and the development of their allogrooming and close proximity networks, as social life is highly important for the welfare of captive chimpanzees [41]. We expected the changes in habitat (from a small cage to a large outdoor enclosure), group size and composition (from two females and one male to three females and four males) to positively affect the welfare of the three chimpanzees, reflected in their activity levels and the respective associations [42]. However, we are aware that assessing welfare is a difficult task [43]. Approaches to conceptualize animal welfare were generally conducted by Duncan and Fraser [44], including the natural behavioural repertoire that is performed. In line with this approach, we would regard the following behavioural changes as positive, i.e., as an indicator for an increased well-being:

- A reduction of abnormal behaviour (if not yet chronically manifested);
- A reduction in the time spent resting and an increase in time spent on locomotion in case of lethargy reflected in *very high* resting and *very low* locomotion values;
- An increase in resting and a decrease in vigilance behaviour in case of *extremely low* resting values and hypervigilance;
- A decline in aggression in case of high amounts of aggressive behaviour;
- A decrease in self-directed behaviours in case of very high values (i.e., indicative of stress);
- A reduction of behaviours typical for immature chimpanzees, such as solitary and social play and object manipulation, in cases of very high values, and/or a replacement by social grooming, a more typical behaviour for adult chimpanzees.

## 2. Materials and Methods

### 2.1. Study Subjects

All three chimpanzees (Table 1) evaluated in this study came from the same owner and arrived at the sanctuary between 2011 and 2012. Before being transferred to the sanctuary, the individuals were housed in two separate, adjacent outdoor cages in the owner’s garden, as part of an animal collection including other mammals and exotic birds.

Coco, a female chimpanzee, was bought from an animal dealer as an infant in Las Ramblas in Barcelona, Spain. Based on investigations and judging from her age at that time, it is most likely that she was caught in the wild in the early 1990s. She was most likely transported in a ship container to the port of Barcelona illegally. Genetic analyses revealed that she belongs to the subspecies *Pan troglodytes troglodytes* originating from populations found in Equatorial Guinea. Initially, she was kept within the owner’s household but was moved to an outdoor cage after becoming troublesome and unpredictable. She remained in close contact with humans for several years.

A few years later another two chimpanzees, Tom (male) and Bea (female) were acquired legally through an intermediary and were housed together with Coco in her outdoor cage. This intermediary obtained them from a circus when they reached the age of 10 and were not of use to the circus anymore. Little information is available regarding their life at the circus other than the legality of both chimpanzees’ documents and the fact that they were always housed together. Genetic analyses revealed them to belong to the subspecies *Pan troglodytes ellioti* originating from populations found in Cameroon. The Spanish laws still permit private collectors, circuses, and trainers to keep chimpanzees if approved CITES (Convention on International Trade in Endangered Species of Wild Fauna and Flora) documents exist for these animals.

While living in these outdoor cages, the owner and employees would regularly interact with the chimpanzees, but not use them in any activities for financial gain. Tom’s owner decided to permanently separate the male from the two females after a few years of living as a group of three, due to his aggressive behaviour and frequent wounding of the females. The three individuals were still able to interact through the adjacent cages, but could no longer harm each other.

Tom was evaluated to be a priority because he was housed singly. He was the first of the three chimpanzees to be transferred to the MONA sanctuary in 2011, and he was integrated into a mixed-sex group. In 2012, Bea and Coco arrived at the sanctuary, and they were integrated into the same mixed-sex group. A few years after arriving at MONA, Bea was diagnosed with severe heart problems. She is now closely monitored and receives medication. 

### 2.2. Study Sites

#### 2.2.1. Living as Pets (Pre-Rescue)

**Figure 1:**
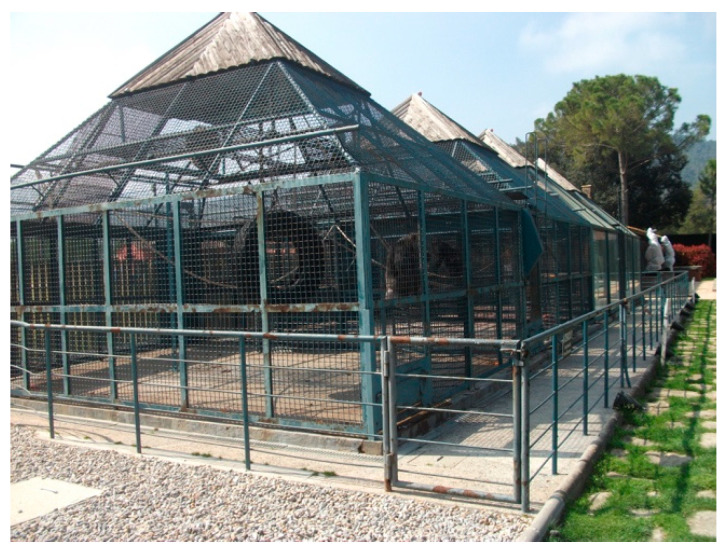
Outdoor cages of the chimpanzees when living as pets.

The first set of data was recorded in 2004 when the three chimpanzees were housed in adjacent outdoor cages. Each of the two cages measured 5.80 m × 4.45 m, with a height of 1.90 m. At the top of each cage, there was a 2.40 m × 1.85 m pyramidal wooden structure with a height of 1.30 m, which served as a shelter in case of bad weather conditions. It was also used to lock up the chimpanzees for maintenance work. Each pyramidal structure was equipped with a heating system (see Figure 1). The cages were furnished with climbing structures and objects such as platforms or wheels. The floor of the cages was made of concrete and there was no access to natural substrate. There were two feeders and drinkers per cage to provide sustenance. Next to the chimpanzees’ cages were other cages housing animals such as birds and other small primates. Dogs were also able to approach the chimpanzee cages. There was no management or care protocol in place. To clean the cages, the keeper used the hose to force the chimpanzees into the pyramidal structure and locked them there. 

The animals’ diets were neither balanced nor species-appropriate and consisted mainly of sweet foods and other unhealthy items. The chimpanzees were fed three times per day. The first feeding was between 7:30 and 8:00 a.m., and consisted of approximately 1 kg of different fruits. The second feeding was between 9:00 a.m. and 1:00 p.m. The third feeding was between 2:00 and 6:00 p.m., and consisted of sweets, dried-fruits, ice-cream, and more fruit. Occasionally the chimpanzees would receive additional treats such as human meals, soft drinks, alcoholic drinks and even cigarettes.

#### 2.2.2. Living at the MONA Sanctuary (Post-Rescue)

The second set of data was recorded between 2020 and 2021 at the MONA sanctuary. The sanctuary is a primate rescue centre located in Girona, Spain. The organisation was a founding member of the European Alliance of Rescue Centres and Sanctuaries (EARS). Since 2000, Fundació MONA has worked in the rescue and rehabilitation of primates who were confiscated from illegal trade activities or were inadequately cared for. Over the first 20 years of existence, MONA has rescued over 30 primates and currently houses two groups of chimpanzees and a group of Barbary macaques. 

During the first few months at the sanctuary, the three chimpanzees went through a habituation and social integration procedure with environmental conditions comparable to their former outdoor cages. Once integrated into the designated mixed-sex group consisting of three adult males and one adult female, the three chimpanzees were moved to the more naturalistic facilities of the sanctuary. The chimpanzee groups were provided with two indoor areas of a size of 25 and 30 m^2^, respectively, and a naturalistic outdoor enclosure with a size of 3.220 m^2^, enriched with a multitude of climbing structures and other artificial structures. All individuals stayed indoors during the night. 

The primates were fed four times per day with a balanced diet based on fruits, seeds and vegetables. At the same time, they were also provided with controlled quantities of other protein-rich foods, and they had access to water ad libitum. A major part of their daily diet was hidden in the naturalistic outdoor enclosure to stimulate foraging and locomotion, as part of their enrichment program. 

### 2.3. Data Collection

The data for the present study were collected at two different locations (see study sites) and times. The same ethogram was used for both study sites. It included the following nine behavioural categories: abnormal behaviour, feeding, locomotion, resting, solitary behaviour, social (interactions with) conspecifics, social interspecific (i.e., social interactions with humans and other animals), vigilance, and other behaviour. The behaviours assigned to the respective behavioural categories and their definitions are provided in the Supplementary Information (Table S1).

The same methodology to collect data was applied at both locations, using instantaneous focal sampling [45] in one-minute intervals during observational sessions of 20 min. Each data set consisted of 30 h of observations per individual and condition, resulting in a total of 180 h of observations used in this study.

The first set of data was collected between July and September 2004 when the three chimpanzees were living as pets (pre-rescue). Data collection was conducted between 8:00 a.m. and 7:00 p.m. and the focal animal was changed after each session. These data were collected using paper and pencil by a bachelor student supervised by Dr. Carmen Maté Garcia, kindly provided by the research team of Barcelona Zoo archives. The bachelor student successfully passed a training program at the Barcelona Zoo and was evaluated as a trained observer after conducting 30 h of valid observations and reaching an 85 percent agreement with the research staff of the zoo.

The second set of data was recorded between November 2020 and April 2021 at the MONA sanctuary (post-rescue). Data collection was conducted between 11:00 a.m. and 5:00 p.m., i.e., while the chimpanzees had access to the outdoor enclosure. Observation sessions were evenly distributed throughout these six hours and the focal animal would change after each session. These data were collected with a tablet device using the behavioural monitoring app ZooMonitor [46,47] by Martí Masip Gimeno (MMG), supervised by MONA’s head researcher Dietmar Crailsheim (DC). MMG was trained by DC over a period of six weeks until reaching an agreement of 85 percent to DC. Data collected during training sessions were not used in the study.

We excluded scans in which the focal animal was either out of sight or the behaviour not clearly recognisable. This led to 3795 focal scans that could be analysed for the pre-rescue condition (Bea: 1175, Coco: 1290, Tom: 1330) and 3785 focal scans for the post-rescue condition (Bea: 1280, Coco: 1163, Tom: 1342). Inter-observer reliability tests between the two observers (i.e., the bachelor student and Martí Masip Gimeno) could not be conducted due to the large time gap between the two observation periods.

### 2.4. Statistical Analysis

The data of each chimpanzee were analysed separately. We compared the individual activity budgets between the pre- and post-rescue condition, i.e., when living as pets vs. when living at the sanctuary, by performing Pearson Chi-Square tests using IBM SPSS Statistics 22. We then evaluated whether the behavioural categories decreased or increased significantly, as indicated by a standardized residual ≥2 or ≤−2. Due to the small sample size (N = 3) in this case study, we did not conduct statistical tests when evaluating the development of certain subcategories such as feeding behaviour, solitary behaviour, and social interactions before and after re-socialisation, but instead described the direction of the development. Additionally, we created allogrooming and close proximity networks for all three chimpanzees for both conditions (pre- vs. post-rescue). Social network graphics were created with the igraph package 0.5.5-351 [48] in R version 4.0.4 [49]. We adapted the R script according to McFarland et al. [50]. In our weighted undirected star networks, ‘vertices’ or ‘nodes’ refer to the individuals, with the focal chimpanzee in the centre and the interaction partners all around. In the case of the close proximity networks, the undirected ‘edges’ represent the percentage of scans the respective dyad spent within an arm’s reach. In the allogrooming networks the undirected ‘edges’ represent the percentage of scans a certain dyad spent on the exchange of allogrooming (i.e., the sum of allogrooming given and received). 

## 3. Results

### 3.1. Activity Budgets

**Figure 2:**
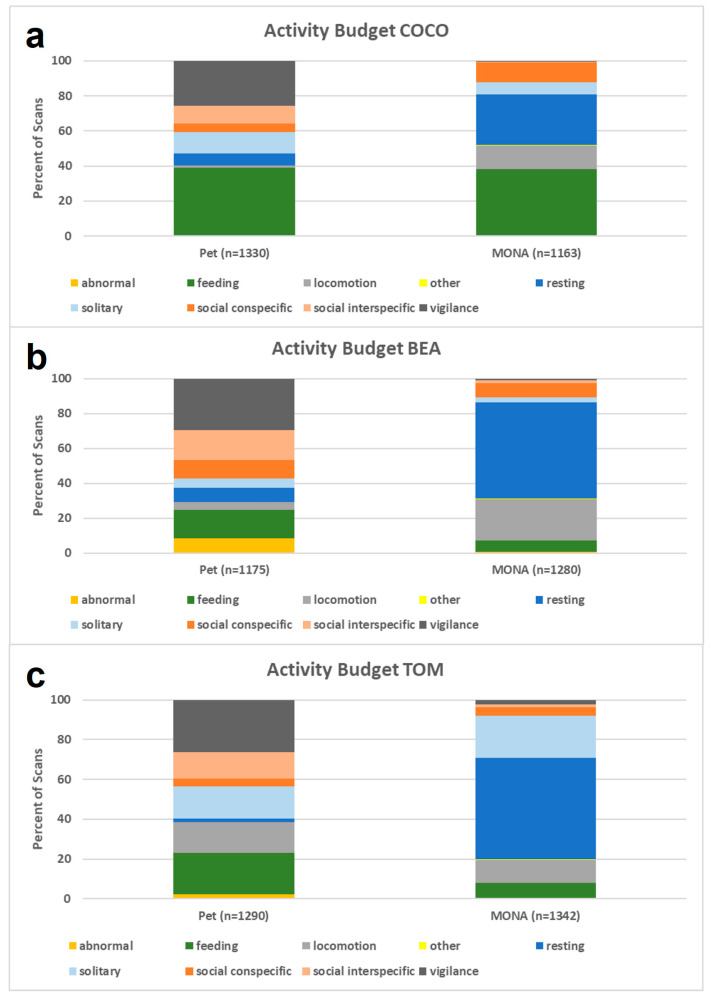
Comparison of the activity budgets while housed as pets (left) and after living at MONA for about 10 years (right) for (**a**) Coco, (**b**) Bea and (**c**) Tom.

We compared the individual activity budgets of two females and one male when kept as pets in 2004, to those in 2020/2021 after living at the MONA sanctuary for around 10 years. The number of scans of the different behavioural categories were compared per individual by using a Pearson Chi-Square Test. The activity budgets (pet condition vs. sanctuary condition) differed significantly in all three individuals (Bea: χ^2^ = 1238.8, df = 8, *p* < 0.001; Coco: χ^2^ = 761.9, df = 8, *p* < 0.001; Tom: χ^2^ = 1106.8, df = 8, *p* < 0.001; see Figure 2).

Bea (8.6% as pet vs. 0.6% at MONA) and Tom’s (2.2% vs. 0.5%) abnormal behaviours decreased significantly in MONA, but were more or less absent in Coco under both conditions (0.1% vs. 0.2%). Bea (16.2% vs. 6.4%) and Tom’s (21.0% vs. 7.6%) feeding behaviours decreased significantly in MONA, but again this pattern was not true for Coco (39.2% vs. 37.9%). A significant increase in locomotion was found with Bea (4.6% vs. 23.8%) and Coco (1.1% vs. 13.6%). By contrast, Tom’s locomotion decreased (15.4% to 11.3%). This, however, may be caused by the fact that pacing, a locomotor stereotype, was not classified as abnormal behaviour but as locomotion while housed as a pet. We do know that Tom was observed pacing during personal observations when visiting the chimpanzees in their former housing, and from the caregiver husbandry information during the first period at MONA. Other behaviours (i.e., defecation and urination) did not change significantly. Resting, which was reduced when kept as pets, increased significantly in all three individuals at MONA (Bea: 8.3% vs. 55.5%, Coco: 6.9% vs. 29.2%, Tom: 1.8% vs. 50.7%). Solitary behaviour (i.e., object manipulation, solitary play, self-directed behaviours) decreased in the two females (Bea: 5.3% vs. 2.7%, Coco: 12.0% vs. 6.6%) but increased in the male, Tom (16.1% vs. 21.4%). The percent of scans spent socially interacting with conspecifics did not change significantly in Bea (10.3% vs. 8.2%) and Tom (4.0% vs. 4.2%) but increased significantly in Coco (4.9% vs. 11.3%). The percentage of scans spent in social interaction with other species decreased significantly in all three individuals (Bea: 17.4% vs. 1.5%, Coco: 10.2% vs. 0.7%, Tom: 13.0% vs. 1.4%). While social interaction with other species included dogs, monkeys, exotic birds, and humans when the chimpanzees were kept as pets, it was only humans at MONA. Vigilance behaviours occurred at an abnormally high level while housed as pets and decreased so significantly they were almost absent at MONA, in all three individuals (Bea: 29.4% vs. 0.9%, Coco: 25.7% vs. 0.3%, Tom: 26.5% vs. 2.3%). In short, the comparison of the activity budgets revealed a significant increase in resting and a substantial decrease in vigilance in all three individuals, which indicates that the chimpanzees became more relaxed after living at the sanctuary. 

**Figure 3:**
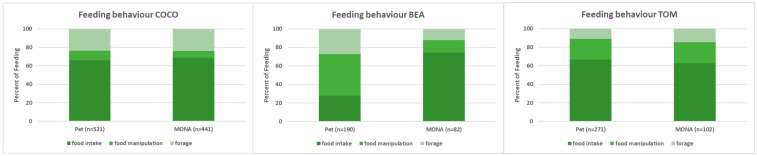
Comparison of the proportions spent on the different subcategories of feeding behaviour while housed as pets and at MONA for Coco (**left**), Bea (**middle**) and Tom (**right**).

When we compared the proportion of time spent on the different subcategories of feeding behaviour—i.e., food intake, food manipulation and foraging (see Figure 3)—we found an increase in the proportion of food intake (27.9% vs. 74.4%) at the expense of food manipulation (44.7% vs. 13.4%) and foraging (27.4% vs. 12.2%) in Bea. Whereas the proportions did not change for Coco and Tom (food intake: Coco: 66.0% vs. 68.9%, Tom: 66.8% vs. 62.7%; food manipulation: Coco: 10.6% vs. 7.3%, Tom: 22.1% vs. 22.5%; forage: Coco: 23.4% vs. 23.8%, Tom: 11.1% vs. 14.7%; Figure 3).

**Figure 4:**
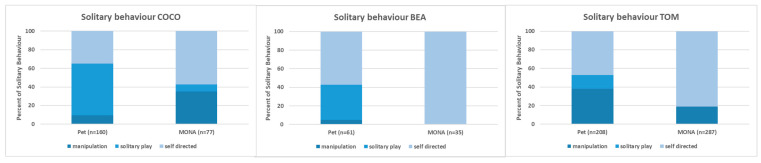
Comparison of the proportions spent on the different subcategories of solitary behaviour while housed as pets and at MONA for Coco (**left**), Bea (**middle**) and Tom (**right**).

The comparison of the proportion of time spent on the subcategories of solitary behaviour (i.e., object manipulation, solitary play, and self-directed behaviour such as autogrooming and inspection of the own body; see Figure 4) revealed a decrease in manipulating objects in Bea (4.9% vs. 0%) and Tom (38.0% vs. 18.8%), but an increase in Coco (9.4% vs. 35.1%). Solitary play decreased or even disappeared in all three individuals (Bea: 37.7% vs. 0%, Coco: 55.6% vs. 7.8%, Tom: 14.9% vs. 0%), whereas the proportion of self-directed behaviour increased for all of them (Bea: 57.4% vs. 100%, Coco: 35.0% vs. 57.1%, Tom: 47.1% vs. 81.2%; Figure 4).

**Figure 5:**
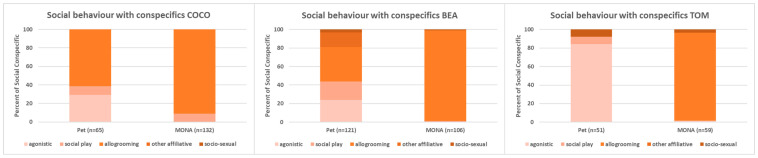
Comparison of the proportions of time spent on the different subcategories of social behaviour with conspecifics while housed as pets, and after living at MONA for about 10 years for Coco (**left**), Bea (**middle**) and Tom (**right**).

Comparing the proportion of time spent on the different subcategories of social interaction with conspecifics (i.e., agonistic interactions, social play, allogrooming, other affiliative interactions and socio-sexual interactions; see Figure 5) revealed that agonistic behaviour disappeared in the two females (Bea: 24.0% vs. 0%, Coco: 29.2% vs. 0%) and noticeably decreased in Tom (84.3% vs. 1.7%). The proportion of time spent on social play decreased in Bea (19.8% vs. 0.9%), disappeared in Tom (7.8% vs. 0%) and did not change in Coco (9.2% vs. 9.1%). Allogrooming became the social behaviour with the largest proportion of time in all three individuals (Bea: 37.2% vs. 98.1%, Coco: 61.5% vs. 90.9%, Tom: 0% vs. 95%). Other affiliative behaviour (i.e., embrace) occurred in Bea only (15.7% when kept as pet). Socio-sexual behaviour decreased in Bea (3.3% vs. 0.9%) and Tom (7.8% vs. 3.3%) and did not occur in Coco at all.

Briefly summarized, it is apparent that allogrooming became the predominant social behaviour in all three chimpanzees after living in a social group for years at the MONA sanctuary. While agonistic interactions reduced significantly or decreased greatly. 

### 3.2. Allogrooming and Close Proximity Networks

**Figure 6:**
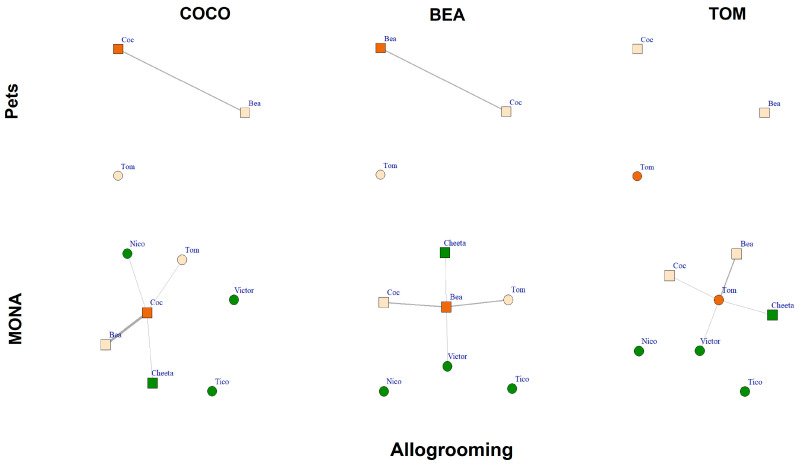
Allogrooming networks of Coco, Bea and Tom while housed as pets (upper row) and at MONA (bottom row). Edges represent percent of scans spent engaged in allogrooming (given and received); circles represent males; squares represent females. Vertex colour: orange = focal individual; peach coloured = familiar individuals; green = new conspecifics at MONA.

The comparison of the allogrooming networks while housed as pets to those when living at MONA for a decade revealed that all three chimpanzees successfully expanded their networks by including new individuals (Figure 6). The two females spent 3.8% (Bea) and 3.0% of scans (Coco), respectively, engaged in allogrooming (i.e., the sum of grooming given and received) when kept as pets, whereas the male Tom was not engaged in allogrooming at all (Figure 6, upper row). At MONA, Bea spent in total 8.0% of scans on allogrooming, of which she spent 3.4% with Coco and 3.4% with Tom. Relating to new group members, Bea spent 1.0% of scans engaged in allogrooming with Cheeta and 0.1% with Victor. Coco spent in total 10.3% of scans allogrooming, of which she spent 7.1% with Bea and 1.8% with Tom. With respect to new group members, Coco spent 0.3% of scans engaged in allogrooming with Cheeta and 1.2% with Nico. Tom spent, in total, 4.3% of scans allogrooming, of which he spent 3.1% with Bea and 0.9% with Coco. Relating to new group members, Tom spent 0.15% of scans engaged in allogrooming with Cheeta and 0.15% with Victor (Figure 6, bottom row). Hence, all three chimpanzees increased their allogrooming network at MONA by including two out of four new conspecifics to their network.

**Figure 7:**
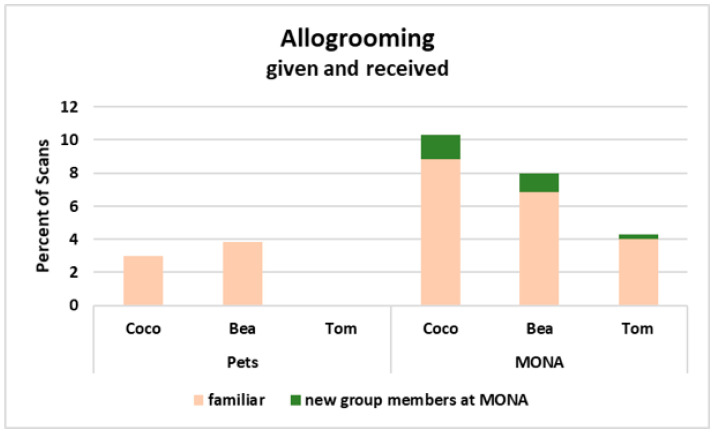
Percent of scans engaged in allogrooming when kept as pets and at MONA for Coco, Bea and Tom (shown as proportion of the total activity). Peach coloured = allogrooming exchanged with familiar conspecifics; green = allogrooming exchanged with new conspecifics at MONA.

Next, we considered the distribution of allogrooming (given and received) between familiar (i.e., among the three individuals who were living together as pets before) and new (i.e., individuals met for the first time at MONA) group members. It became obvious, that all three individuals increased the percentage of time spent on allogrooming in total, however, a major part of allogrooming was still directed towards familiar conspecifics at MONA, and only very small parts were directed to two out of four new group members (Figure 7). 

**Figure 8:**
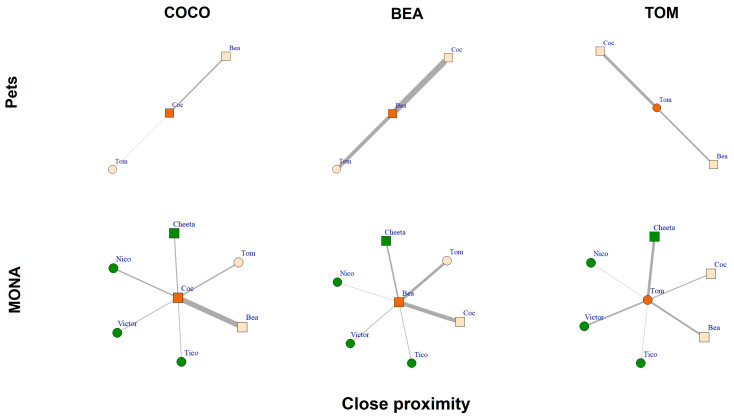
Close proximity networks of Coco, Bea and Tom while housed as pets (upper row) and at MONA (bottom row). Edges represent percent of scans spent within close proximity (i.e., within an arm’s reach); circles represent males, squares represent females. Vertex colour: orange = focal individual; peach coloured = familiar individuals; green = new conspecifics at MONA.

Comparing the close proximity networks showed that all three chimpanzees spent some time within an arm’s reach of their group members when kept as pets and at MONA (Figure 8). As pets, Bea spent a total of 35.5% of scans in close proximity to her conspecifics (21.5% to Coco and 14.0% to Tom). For Coco and Tom, 6.2% of scans (5.2% to Bea and 1.1% to Tom) and 17.7% of scans (6.9% to Bea and 10.8% to Coco; Figure 8, upper row) were in close proximity of conspecifics, respectively. Comparatively, at MONA, Bea spent 31.5% of scans in close proximity to her group members (ranging from 0.5% to Nico to 13.4% to Coco). Coco spent 33.4% of scans in close proximity to her group members (ranging from 2.3% to Victor to 16.8% to Bea) and Tom spent 24.3% of scans within an arm’s reach of his conspecifics (ranging from 0.1% to Tico to 8.6% to Cheeta; Figure 8, bottom row). The percentage of scans spent within close proximity of others remained consistently high for Bea across both conditions. However, this behaviour strongly increased in Coco and Tom when in MONA. While Bea and Coco showed a preference to be close to one other, Tom preferred to be within close proximity of the female Cheeta. 

Overall, we show that all three chimpanzees increased their close proximity networks by spending time within an arm’s reach of all other group members at MONA. This finding, together with the results of negligibly low or no occurrence of agonistic interactions provides an indication of the optimal appropriate group composition at MONA.

## 4. Discussion

In this case study, we had the rare opportunity to access behavioural data of three chimpanzees, while they were still housed in a species-inadequate environment as pets, six years before being rescued and transported to a primate sanctuary. By collecting data on the same individuals approximately 16 years later, when rehabilitated and integrated into a social group of conspecifics at the sanctuary for approximately 10 years, we were able to compare their activity budgets individually between those two conditions. The main objective was to determine whether species-typical behaviours relating to positive welfare such as allogrooming and time spent in close proximity to their conspecifics would increase, while undesired behaviours such as abnormal behaviour, hypervigilance and high levels of aggression, would decrease, or could even be eliminated. Furthermore, we aimed to compare the chimpanzees’ social capacities before and after rescue, following their integration within their newly established social group.

As expected, the results showed that the time spent on certain behaviours changed substantially between pre- and post-rescue, which may be a result of changes in habitat and the social environment in terms of new group members. Yet, the fact that there were also some differences between the three individuals highlights the importance of examining and evaluating each chimpanzee individually, rather than reporting average values for all three individuals together. The differences found were mostly in directions which were desired by the sanctuaries’ rehabilitation objectives: we found an increase in resting and a simultaneous decrease in vigilance in all three chimpanzees. This suggests that resting replaced time spent on vigilance behaviours at the sanctuary, compared to when living as pets. Moreover, at the sanctuary, the chimpanzees were more socially competent and age-appropriate, as social play decreased strongly, while allogrooming became the predominant type of social interaction and agonistic interactions almost disappeared completely. Furthermore, all three chimpanzees successfully expanded their allogrooming as well as close proximity networks at the sanctuary, by including new group members as social partners. Although, as expected, all three subjects maintained their closest relationships with familiar individuals, i.e., with each other. These findings suggest that the sanctuary environment and the social group composition seem to be appropriate, contributing to the individuals’ well-being.

There are few publications that compare the lives of chimpanzees when living in species-inadequate conditions to those living in improved conditions later on. This is because such data are usually not available. Obtaining trustworthy data and information regarding the past lives of former pet and entertainment chimpanzees is seldom possible. Even if obtained, these data may have been influenced by diverse individual factors such as sex, age, degree of human exposure, housing conditions, care conditions, early life experience, etc., which make a comparison difficult.

Based on the information collected by the sanctuary and provided by the previous owner, all three chimpanzees were caught from the wild, were maternally deprived in their early infancy, and were human reared. Furthermore, all three individuals were lacking species-appropriate care and adequate housing facilities in accordance with their needs. Thus, although at different times of their lives and in different settings, all three chimpanzees experienced traumatic life events and experienced adverse living conditions, described in the literature as being detrimental to the development of social competence and well-being [2,7,9,10,51,52,53,54]. These adverse experiences include the trauma of being caught in the wild, and a dramatic change in living conditions [2,7], as well as experiencing maternal deprivation, lacking social partners [51,52,53,54], and being exploited for financial gain for personal entertainment in circuses and/or as pets [9,10]. All three chimpanzees arrived at the sanctuary as adults, and thus were exposed to these conditions throughout their immature life (i.e., from early infancy onwards). As such, it is expected that their early socio-emotional development and their capacity to cope with the environment as reflected in their social competence were strongly influenced by these adverse living conditions and traumatic experiences. It has been shown that impairments in social competence are most pronounced in wild-caught, severely deprived former laboratory chimpanzees [55]. However, they were also found in wild-caught former pet and entertainment chimpanzees [11] and in wild-caught but socially reared zoo chimpanzees [28]. Several other studies also suggest that early life adversities and a high degree of human exposure during the rearing age of chimpanzees are associated with social behavioural deficiencies, stereotypic behaviours and altered long-term brain structure [56,57,58,59,60,61,62].

Behaviour can be used as a welfare indicator, as it informs not only about the individual’s internal physiological state, but also reflects responses to the external environment [63]. In other words, the occurrence, frequency, and duration of species-specific as well as abnormal behaviours can serve as an indicator of welfare in captive animals [64,65,66]. For example, the occurrence of abnormal behaviours might originate from past adverse living conditions, but could also point to specific stressful situations [66,67,68]. Providing appropriate welfare is a major concern of sanctuaries, because it requires the capacity to detect and comprehend factors or conditions that may have a major impact on the animals’ well-being, both for the better and the worse [69]. One approach to assess welfare is by comparing the activity budgets of free-living and captive chimpanzees [70,71]. However, these comparisons must be interpreted with caution, because deviations may not reflect decreased welfare [72], and may instead be caused by ecological constraints [73]. The complexities and obstacles of these comparisons are explained in detail in Howell and Cheyne [74]. Therefore, we feel that comparing the activity budgets of our former pet chimpanzees to other captive populations is more appropriate. We were able to study how the different behaviours of the study subjects changed after living in a larger social group at the sanctuary.

Table 2 provides a comparison of the activity budgets of captive chimpanzees with different rearing backgrounds to those of our chimpanzees. 

Feeding behaviour ranged between 10 and 30 percent in other captive populations [36,70,71] and our chimpanzees fell within this range when kept as pets and also after living at the sanctuary. Interestingly, feeding did decrease in the human-reared chimpanzees after their relocation to a bigger enclosure [36]. This corresponded to the pattern we found in our chimpanzees after their relocation to the sanctuary. Resting ranged between 41 and 70 percent in the other populations of captive chimpanzees [36,70,71]. Compared to these values, resting was reduced significantly in our chimpanzees when kept as pets. However, after living at the sanctuary for about ten years, the resting values of our chimpanzees fell within this range. Regarding locomotion, we found our chimpanzees to be within the range of the other captive populations (range 5–12 percent) while kept as pets. Those locomotion values, however, were exceeded by our chimpanzees when living at the sanctuary. The time spent on solitary behaviours are comparable between the human-raised chimpanzees in Washington and our chimpanzees under both housing conditions. However, there was a slight decrease in both populations after being relocated. While the stereotypic (abnormal) behaviours were low in the human-reared chimpanzees at Washington under both housing conditions, it was higher in our chimpanzees while kept as pets, and decreased to comparable values when living at the sanctuary. There was a huge variability among the individuals housed at the Tama Zoological Park in Japan, regarding time spent on social grooming. Our chimpanzees were at the lower limit when housed as pets. However, they showed on average a more than three-fold increase in their time spent allogrooming when living at the sanctuary. If all social interactions with conspecifics (including affiliative as well as agonistic behaviour) were taken into account when comparing the human-reared chimpanzees at Washington and our chimpanzees, it became apparent that our chimpanzees were interacting with their group conspecifics half as much as those at Washington, on average.

This relatively high amount of time our chimpanzees spent feeding when kept as pets may have been due to the fact that the chimpanzees often received snacks during the day, and therefore spent a major portion of their time feeding. Bea was the exception, who spent more time with food manipulation when kept as a pet. Time spent feeding decreased substantially in the two older individuals, Bea and Tom, when living at the sanctuary, even though most of the food is provided hidden and distributed in the outside enclosure. One reason for this may be their older age (about 36 years), since Coco, the younger female spent about 38 percent of time feeding when living at the sanctuary. Furthermore, the reduction may be partly explained by the fact that the chimpanzees receive their evening feed at MONA indoors; hence, it is not included in the activity budget for which data were collected outdoors.

Time spent resting was reduced significantly in all three individuals while living as pets. However, time spent resting may have been underestimated because the individuals were out of sight when staying within the pyramidal structure of their cages. Simultaneously, however, the values for vigilance were extraordinarily high, ranging from 26 to 29 percent of time. These very high values may have been indicative of hypervigilance, as described by Bradshaw et al. [75] and Lopresti-Goodman et al. [76] as observed in distressed former laboratory chimpanzees. Hypervigilance may have been caused by the chimpanzees being kept in a cage in the garden of a private house, where many people were coming in and out. This pattern was reversed in all three individuals after living at MONA for about ten years. While time spent resting increased significantly, vigilance almost disappeared, occurring in only 0.3 to 2 percent of scans. The increase in resting in the two older individuals, Bea and Tom, might also be partly due to their increased age. We consider this simultaneous increase in resting and decrease in vigilance as a positive behavioural development.

Time spent on locomotion was very low in the two females when kept as pets; however, it was significantly higher for the male chimpanzee, Tom. This higher value found for Tom is most probably due to the fact that he exhibited stereotypic pacing when living in the small cage before his rescue. This was observed incorrectly as locomotion and was not considered an abnormal behaviour. Tom showed stereotypic pacing early on at MONA as well, but it has now completely disappeared. However, locomotion increased substantially in the two females when living at the sanctuary, and only slightly decreased in Tom, despite the fact that he was not pacing anymore. This might be the result of having access to a large outdoor enclosure now. This increase in locomotion is also considered a positive behavioural development.

Abnormal behaviours did occur in Bea and Tom when kept as pets, but were rarely present in Coco. Bea, Coco and Tom exhibited stereotypic biting of the fence, but this disappeared after their arrival at the sanctuary. Additionally, Bea showed further unspecified stereotypic behaviours when living as a pet. Abnormal behaviours were more or less absent in all three individuals a decade after their arrival at MONA. There were rare occurrences of coprophagy in Coco and Tom and of overgrooming in Bea and Tom. Coco showed two more frequent abnormal behaviours: stereotypic self-scratching and self-poking. However, as she did not harm herself and the behaviour was exhibited during her normal daily activity, i.e., during resting, feeding and allogrooming, we considered these two behaviours as tics, and did not count them as abnormal. The decrease in abnormal behaviour in Bea and Tom is a further positive behavioural development.

Overall, solitary behaviour decreased in the two females but increased in the male chimpanzee at MONA, compared to when living as pets. With respect to the different behaviours assigned to solitary behaviour, we found the proportion of time spent in solitary play to decrease, or even disappear, whereas self-directed behaviour increased for all three individuals at the sanctuary. If we compare the averaged solitary behaviours (including solitary play, object manipulation and autogrooming) to that of the chimpanzees observed by Jensvold et al. [36], the values are similar, but there is a marginal decrease in both populations after relocation to the sanctuary. The behavioural development is valued positively for the two females, as their time spent exhibiting solitary behaviour decreased, but not for Tom, whose values for self-directed behaviour (autogrooming) remained high. This may be an indicator for exposure to occasional stress [77]. 

While all three individuals spent certain amounts of time interacting with humans and other animals when kept as pets, these interspecific interactions almost disappeared at the sanctuary. The few occasions of observing interspecific interactions at the sanctuary were directed towards caregivers while providing food or working in the surrounding enclosure. 

We were interested to observe the development of social interactions with conspecifics, because we expected social competence to be impaired in these former pet chimpanzees due to their inadequate early life experiences. While the time spent socially interacting with conspecifics did not change significantly in the two older individuals, Bea and Tom, it increased significantly in the younger female Coco. In addition, agonistic behaviours were rarely present in all three individuals at MONA. The portion of time spent on social play activities did not change in the younger female Coco. However, social play was almost absent for the two older individuals, which is in line with studies on captive and free-living chimpanzees that show social play occurs rarely among adult individuals [78,79]. This decrease in aggression, together with an increase in social grooming, is considered a positive behavioural development.

One of our most important findings was that allogrooming became the predominant social behaviour at the sanctuary. Allogrooming is of utmost importance, not only in terms of its hygienic function but also in enabling the building of new relationships, and the maintenance of already established relationships. It also supports the formation of bonds, as well as coalitions among group members [15,26,80,81]. We found a marked increase in allogrooming in the two females from being kept as pets to living at MONA. Allogrooming in the two females doubled and tripled, respectively. In contrast, the male chimpanzee Tom was not involved in allogrooming at all while living as a pet, but he engaged in allogrooming more than 4 percent of the time at the sanctuary. This increase in time spent allogrooming, as well as the extension of the allogrooming network provides an indication of improved social competence in all three individuals. We previously mentioned that allogrooming was exchanged with new group members only rarely. It still mainly occurred among the three familiar individuals, showing that mutual friends are more likely to groom each other [82]. With respect to their close proximity networks, all three individuals spent at least some time within an arm’s reach of all of their group members, and did not avoid their proximity. Thus, they increased their close proximity networks at MONA as well. All three chimpanzees exchanged grooming with four out of six possible partners. However, with regard to close proximity, they included all group members. The finding that all three chimpanzees were more constrained with respect to allogrooming fits with previous findings for maternally deprived wild-caught chimpanzees who became socially reared zoo chimpanzees [28]. 

It is important to note that three individuals comprise a very small sample size, and the findings should be treated with caution. However, we do think that it is worth being reported. Access to this type of information is rarely available. 

In conclusion, the overall findings suggest that all three individuals recovered after living for about ten years at the sanctuary. We also found that resting increased to amounts comparable with other captive populations without adverse rearing histories. The abnormally high levels of vigilance behaviour displayed previously also declined sharply. Furthermore, social competence improved in all three chimpanzees, indicated by: allogrooming becoming the predominant social behaviour; agonistic interactions diminishing; and allogrooming and close proximity networks extending by including new group members. 

## 5. Conclusions

From all these findings, we observed that the well-being of these three individuals, namely Bea, Coco and Tom, increased a decade after arrival at the MONA sanctuary. Many other zoos and sanctuaries have also demonstrated that with careful management, former pet and performer chimpanzees can be integrated successfully into functioning social groups, despite challenges with socio-behavioural development, and are able to benefit from the social companionship of conspecifics [56].

Chimpanzees are long-living, highly social beings [83,84] that have not evolved to be pets or performers. The treatment and conditions these chimpanzees endured leave scars for the rest of their lives. Retiring them to sanctuaries is the best option we can provide them.

## Figures and Tables

**Table 1:** Biographic information on the study subjects.

Name	ID	Sex	Origin	Subspecies	Previous Experience	Est. Year of Birth	Arrival at MONA
COCO	COC	F	Wild-caught	*P. t. troglodytes*	Pet from mid-1990s	1994	2012
BEA	BEA	F	Wild-caught	*P. t. ellioti*	Circus since mid-1980s, Pet from mid-1990s	1985	2012
TOM	TOM	M	Wild-caught	*P. t. ellioti*	Circus since mid-1980s, Pet from mid-1990s	1985	2011

**Table 2:** Comparison of the activity budgets of our three chimpanzees to other captive chimpanzees (Tama Zoological Park [70] and Primate Research Institute (PRI) [71] in Japan; Central Washington University in Ellensburg [36]). Numbers refer to percentage of observation time.

	Tama Zoologcial Park Japan (N = 16)	PRI Japan (N = 12) *		Human-Raised Chimpanzees Washington, USA (N = 5)		MONA Chimpanzees (N = 3)	
Behaviour: Percent of Time Mean (± SD) and/or [Range]		Cognitive Experi-ment (N = 6)	No Partici-pation in cog. exp. (N = 6)	Small Enclosure (PBF)	Bigger Enclosure (CHCI)	Housed as Pets	At the Sanctuary
Feeding (including Foraging)	18.8 (± 7.4)	30	10	23.1	14.9	25.5 (± 12.1) [16.2–39.2]	17.3 (± 17.9) [6.4–37.9]
Resting	50 [40,41,42,43,44,45,46,47,48,49,50,51,52,53,54,55,56,57,58,59,60,61,62,63,64,65]	45	70	41.1	49.4	5.7 (± 3.4) [1.8–8.3]	45.1 (± 14.0) [29.2–55.5]
Locomotion	12.0 (± 3.6)	10	7	5.0	8.8	7.0 (± 7.5) [1.1–15.4]	16.2 (± 6.7) [11.3–23.8]
Solitary behaviour (solitary play, object manipulation, self-groom)	--	--	--	9.6	8.6	11.1 (± 5.5) [5.3–16.1]	10.2 (± 9.9) [2.7–21.4]
Stereotypic	--	--	--	0.6	0.3	3.6 (± 4.4) [0.1–8.6]	0.4 (± 0.2) [0.2–0.6]
Social grooming	[2,3,4,5,6,7,8,9,10,11,12,13,14,15,16,17,18,19,20]	--	--	--	--	2.3 (± 2.0) [0–3.8]	7.5 (± 3.0) [4.3–10.3]
Social interaction with conspecifics (affiliative and agonistic)	--	--	--	17.5	14.1	6.4 (± 3.4) [4.0–10.3]	7.9 (± 3.6) [4.2–11.3]

Abbreviations: BPF = Psychology Building Facility; CHCI = Chimpanzee and Human Communication Institute. -- = No data available. * Six out of twelve chimpanzees participated in cognitive experiments and six chimpanzees did not participate in these experiments.

## Data Availability

All data are reported in the Results section. Additional information can be obtained from the corresponding authors upon request.

## Supplementary Materials

The following supporting information can be downloaded at: https://www.mdpi.com/article/10.3390/ani12020138/s1, Table S1: Ethogram.
