# Supplementary material for: Behavioural Development of Three Former Pet Chimpanzees a Decade after Arrival at the MONA Sanctuary
Source: Animals (Basel). 2022 Jan 7;12(2):138. doi: 10.3390/ani12020138 (PMC8772579; doi:10.3390/ani12020138)
Supplement: Supplementary file 1 [file animals-12-00138-s001.zip › animals-1496086-supplementary.pdf]

Behavioural Development of Three Former Pet Chimpanzees a Decade after Arrival at the MONA Sanctuary

Feliu et al. (2022)

Supplementary Information

Table S1: Ethogram

	**Behavioural Category**	**Behaviours**	**Definition**
Behaviour	Abnormal behaviour	Pacing*	Repetitive and invariant movement on the same path for no apparent reason
		Overgrooming	Repetitive and invariant body cleaning behaviour directed to a specific area of own body with fingers or mouth including pulling out hair
		Coprophagy	Ingestion and manipulation of faeces with mouth
		Fence biting	Repetitve and invariant biting of the fence
	Feeding	Food intake	Intake of food and liquids
		Forage	Searching for and localisation of food
		Food manipulation	Hoarding and transport of food; playing with food; inspection of feeding enrichment
	Locomotion		Moving (horizontal) or climbing (vertical)
	Resting		Sitting, lying, dozing, or sleeping
	Solitary	Object manipulation	Inspection of objects of the environment and non-food enrichment with hands and/or feet
		Solitary play	Solitary motor play including swinging, hanging, jumping, pivoting, sliding;<br>object play when carrying or using object(s) accompanied by laughing and showing a play face
		Self-directed	Inspection of own body,<br>autogrooming with fingers and/or mouth
	Social conspecific	Agonistic behaviour	Aggression with body contact including hits, bites, fighting and scratching; aggression without body contact including threats and chasing;<br>display with piloerection, including running, jumping, hitting objects, body swaying and vocalizations;<br>submissive behaviour including fleeing and screaming;<br>displacement
		Social play	Play chase where one chases the other individual;<br>rough-and-tumble play including hitting and pushing;<br>muzzling with gentle bites;<br>holding hands where the playing individuals touch their hands;<br>social object play;<br>often accompanied by play face and laughing
		Allogrooming	Body-cleansing behaviour from one individual towards another individual with fingers and/or mouth, includes mutual grooming; grooming request
		Other affiliative behaviour	Touching, embracing another individual, hold out hand
		Socio-sexual behaviour	Genital inspection includes the manipulation of the genitals by visual, olfactory or phyisical inspection;<br>genital presentation where the female presents her anogenital region towards the male; copulation

	Social interspecific	Affiliative and agonistic interactions with human and/or other animals	Request for interaction or affiliative interactions with humans (keepers, observers, visitors) and/or other animals (dogs, horses, macaques); display towards humans (keepers, observers, visitors) and/or other animals (dogs, horses, macaques)
	Vigilance		Scanning of or starring at the environment while sitting or standing in a tight body posture
	Other	Excretion	Defecate/Urinate
		Masturbation	Self stimulation of the genitals with hand and/or mouth
Association	Close proximity		Staying in body contact up to an arm's length to another individual without interacting

* Please note that Pacing has been assigned to Locomotion during the pre-rescue period (i.e., when living as pets).
